# The Biodiversity Informatics Potential Index

**DOI:** 10.1186/1471-2105-12-S15-S4

**Published:** 2011-12-15

**Authors:** Arturo H Ariño, Vishwas Chavan, Nick King

**Affiliations:** 1 Department of Zoology and Ecology, University of Navarra, E-31080 Pamplona, Spain; 2 Global Biodiversity Information Facility Secretariat, Universitetsparken 15, DK 2100, Copenhagen, Denmark

## Abstract

**Background:**

Biodiversity informatics is a relatively new discipline extending computer science in the context of biodiversity data, and its development to date has not been uniform throughout the world. Digitizing effort and capacity building are costly, and ways should be found to prioritize them rationally. The proposed 'Biodiversity Informatics Potential (BIP) Index' seeks to fulfill such a prioritization role. We propose that the potential for biodiversity informatics be assessed through three concepts: (a) the intrinsic biodiversity potential (the biological richness or ecological diversity) of a country; (b) the capacity of the country to generate biodiversity data records; and (c) the availability of technical infrastructure in a country for managing and publishing such records.

**Methods:**

Broadly, the techniques used to construct the BIP Index were rank correlation, multiple regression analysis, principal components analysis and optimization by linear programming. We built the BIP Index by finding a parsimonious set of country-level human, economic and environmental variables that best predicted the availability of primary biodiversity data accessible through the Global Biodiversity Information Facility (GBIF) network, and constructing an optimized model with these variables. The model was then applied to all countries for which sufficient data existed, to obtain a score for each country. Countries were ranked according to that score.

**Results:**

Many of the current GBIF participants ranked highly in the BIP Index, although some of them seemed not to have realized their biodiversity informatics potential. The BIP Index attributed low ranking to most non-participant countries; however, a few of them scored highly, suggesting that these would be high-return new participants if encouraged to contribute towards the GBIF mission of free and open access to biodiversity data.

**Conclusions:**

The BIP Index could potentially help in (a) identifying countries most likely to contribute to filling gaps in digitized biodiversity data; (b) assisting countries potentially in need (for example mega-diverse) to mobilize resources and collect data that could be used in decision-making; and (c) allowing identification of which biodiversity informatics-resourced countries could afford to assist countries lacking in biodiversity informatics capacity, and which data-rich countries should benefit most from such help.

## Background

### Idea and rationale

Progress in biodiversity informatics (methodologies and tools extending contemporary computer science and informatics principles in the context of biodiversity data [1]) is not homogeneous throughout the world, with the differences apparently due more to the economic status of countries than to their estimated biodiversity richness [2], as is the case for data availability in literature [3]. Digitizing all available data already existing in analog form or locked in unavailable databases has been shown to be impractical [2,4,5]. Therefore, digitizing efforts, related informatics infrastructure development and capacity building, being limited, should be both prioritized and encouraged.

The BIP Index seeks to fulfill a prioritization role, by integrating a number of parameters that might be related to the state of biodiversity informatics in individual countries. It could potentially:

(a) help identify countries or economies most likely to be able to contribute to filling gaps in digitized data, as well as being most likely to absorb, implement and reliably build required informatics infrastructure and capacity in biodiversity informatics;

(b) provide a prioritization mechanism, by integrating a number of parameters that might be related to the state of biodiversity informatics in individual countries: infrastructure capacity (financial, human and technical resources), data accessibility, and fitness for use of accessible data;

(c) help countries, especially those with the most need (for example mega-diverse countries, or those whose biodiversity is most endangered), to mobilize resources and collect data that could be used in decision-making; and

(d) be used as an equalizing measure involved in any biodiversity informatics compensation mechanisms across countries; for instance, the BIP Index might allow identification of countries with a high level of biodiversity informatics resources that could afford to invest some of those resources in countries lacking them, in an efficient way that would be most likely to produce useful, quality data after initial capacity building.

### Definitions

**Figure 1:**
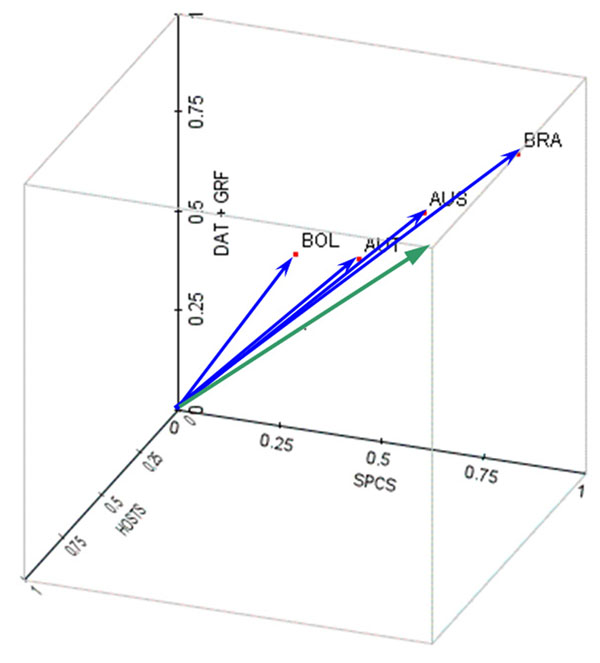
A graphical representation of the concepts in the BIP Index, and example for four countries. The BIP Index for a country is the Euclidean distance to origin in a four-dimensional space (here represented as a three-dimensional space for simplicity; two of the dimensions have been merged together in the *z* axis). The dimensions of the space represent the capacity of the country to hold biodiversity data (SPCS), related to concept 1 in the Definitions section; to generate raw biodiversity data (DAT) or quality biodiversity data (GRF), related to concept 2; and to host biodiversity data (HOST), related to concept 3. The green vector signals how BIP increases along the three concept scales: the higher a country ranks against these concepts, the greater its BIP score and therefore its biodiversity informatics potential. Thus, a country occupies a position in this space, and the length of the vector from the origin to the country's position (its Euclidean distance) is the BIP Index. The longer the vector, the higher the BIP score. The highest possible BIP Index is the length of the green vector. The four blue vectors are the BIP Index scores for four example countries. A country can be nearer one plane than other country, meaning that that dimension is more important in that country. For example, Brazil (BRA) has higher potential than Australia (AUS) or Austria (AUT) mainly because of higher biodiversity potential, and these two countries, also with a high BIP Index, owe it more to their hosting capacity. Bolivia (BOL) also lies towards the DAT+GRF and SPCS planes (more so to the latter), but has a lower score and thus a lower overall BIP Index.

The state of biodiversity informatics for a country is defined here as a composite of three concepts (Figure 1):

1. The **intrinsic biodiversity potential** of a country (broadly, its biological or ecological richness and factors favoring it), related to its physical, biological and environmental characteristics.

2. The capacity of the country to **generate biodiversity data records**, related to its intrinsic biodiversity potential and to its ability to disclose such potential through data records. This data generation, in turn, contains two related but distinct components:

a. The raw data generation potential, producing basic data records (specimens, samples, observations), and

b. The quality data generation potential, producing biodiversity value-added records by generating additional data enhancing their fitness for use.

3. The availability of **technical infrastructure** in a country for hosting, managing and sharing biodiversity data records, both produced in the country as a result of its own biodiversity potential and data generation capacity, or existing in the country as a result of research efforts directed towards other countries.

These three concepts can be further summarized along two main orthogonal axes:

i. The capacity to generate primary biodiversity data, and

ii. The capacity to discover, curate and make available such data for public access.

In this context,

• Primary biodiversity data are documented events manifesting the **occurrence** of an identified biological entity in a definite space and time;

• Primary biodiversity data are atomized into primary biodiversity records (PBRs) that can be hosted by the country generating them, or by any other country; and

• 'Hosting' here means that a facility in a country makes the PBRs accessible to any interested party, following the principles of free and open access to data.

With these definitions in mind, the BIP Index is a composite of a number of country-level indicator variables (data, statistics or indexes representing any measurable, scalable or ordered concept that are available as a single measure for a country) that can predict the state of biodiversity informatics in countries.

## Methods

### Development of BIP Index: overview

**Figure 2:**
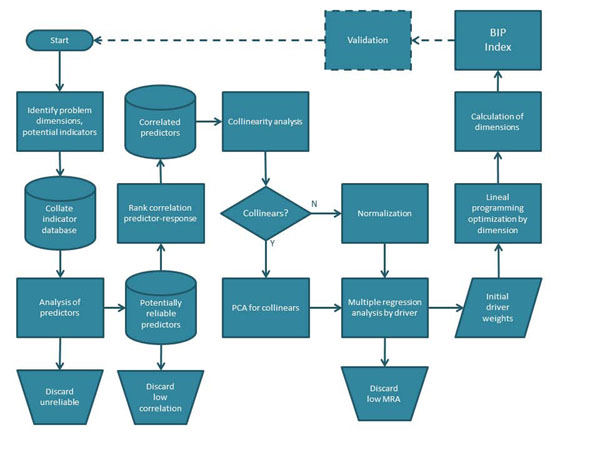
A general flow chart of the BIP Index development cycle and statistical approach. Dashed line represents a future flow to be repeated periodically. MRA: step-wise multiple regression analysis.

The BIP Index sought was a single scale against which countries could be ranked according to their potential to invest in, strengthen and benefit from biodiversity informatics. However, the BIP Index could itself be composed of sub-indexes, each representing one aspect of the general BIP Index idea, and BIP Index development can thus proceeded analytically. Decomposed into concepts (as described above), these concepts, in turn, were decomposed until groups of elemental predictors (country-level variables) could be found. Once predictor variables and response variables were identified, a BIP Index could be constructed as a model that related predictor to response variables (Figure 2).

### BIP Index construction

**Dimensions.** To identify adequate variables, some response variables or known proxies for the state of biodiversity informatics were needed. Predictor variables could then be compared with the proxies if cases could be found, and a general model could be derived to be applicable to the remainder countries.

The chosen proxies were the number of records made available through GBIF's index [8]. GBIF can be regarded as a sample of the world's biodiversity knowledge as represented by PBRs [6]. Four sets of data were available related to hosting and generation of PBRs. These sets represent the 'dimensions' of the BIP Index, related to its ability to predict data generation and data hosting by countries. The four dimensions were:

(a) Number of PBRs occurring in each country (whether published by that same country or by another country), hereinafter DAT, as an indicator of the potential raw biodiversity data produced in that country.

(b) Number of geo-referenced PBRs occurring in each country (whether published by that same country or by another country), hereinafter GRF, as an indicator of the higher quality biodiversity data produced in that country.

(c) Number of PBRs made public by a country (whether occurring in that same country or in another country), hereinafter HOST, as an indicator of the technical hosting capacity of that country.

(d) Number of different taxa, generally at the species level, listed in the PBRs occurring in a country, hereinafter SPCS, as an indicator of the potential raw biodiversity data existing in that country.

The BIP Index is a composite of predictions for these four dimensions based on the predictor variables, tested against these dimensions known from current GBIF participant countries.

DAT and GRF are closely related variables (GRF being a subset of DAT) and in the final BIP Index formulation, these two dimensions are weighted and amalgamated into one, yielding the three-dimensional vector that forms the current version of the BIP Index. Further, SPCS can be combined with the DAT-GRF dimension into the 'data generation' axis, theoretically orthogonal (but not uncorrelated) to the 'data hosting' axis represented by HOST. In theory, a country with rich biodiversity (SPCS) and large biomass-related size (DAT-GRF) should have a higher potential to produce biodiversity data, other parameters being equal.

**Predictor variables.** The BIP Index attempts to explain the response variables from a relatively small set of meaningful predictor variables. Thus, much of the work in developing the BIP Index was choosing which predictors, from many available, would contribute to the formulation of the BIP Index and which predictors would have little or no predicting power and could be discarded.

The predictor variables could belong to at least three main areas that concur into the BIP Index:

(a) Economic power indicators, which may underlie efforts at directing resources towards research and obtaining data. These can in turn be related to sociological indicators, as well as raw power. Example indicators are: gross domestic product (GDP), purchasing power parity (PPP), per-capita income (PCI) and economic models; geographical indicators such as size and exclusive economic zone (EEZ); social indicators such as population, percentage literacy, percentage employment and Gini coefficient.

(b) Data potential indicators. Biodiversity richness, as measured through appropriate proxies that may result in data: higher biodiversity or larger relative natural areas might mean more potential data. Conversely, reduced biodiversity through soil use may reduce data expectation. Example parameters are: species richness and diversity, hotspots, ecological footprint, number of endemic species and number of collections.

(c) Informatics capacity. The data availability can be enhanced by power, but the databasing and sharing depends on information technology capacity. Example indicators are: digital opportunity index (DOI), educational level and bandwidth per capita.

**Figure 3:**
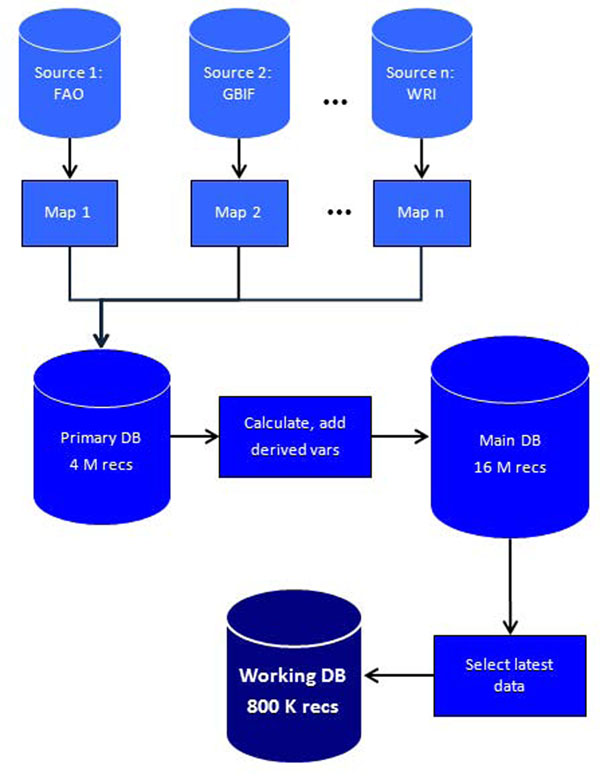
Preparation of the variable database. Datasets obtained from various sources (such as the Food and Agriculture Organization of the United Nations (FAO) [7], GBIF [8] and WRI [17]) were mapped to a common structure. Derived variables were treated as new variables. Time series of datasets were retained, but only the latest available data were used for the working database. DB: Database; recs: records; vars, variables.

The predictor variables were selected from sets of publicly available, country-level, year-specific variables from a number of fields, including biological, developmental, financial and infrastructure. A database of available variables was constructed to allow homogeneous analysis (Figure 3).

Many predictor variables were naturally correlated with intrinsic country variables related to its 'size'. For instance, the total amount of parkland surface in a large country could naturally be larger than that of a smaller country. Therefore, those variables that would acquire a different meaning when taking into account some basic feature of the country were relativized into derived variables, by dividing them according the country's size, population, or gross domestic product (GDP) variables. Some variables with skewed distributions were also log-normalized. Derived variables were added to the database.

The roster of potential predictors thus included approximately 5,000 variables: more than 1,300 primary variables collated from public sources and nearly 3,700 variables derived from the primary variables after relativization for about 250 countries, belonging to the three main categories. Within categories, specific subsets of variables constituted the nine drivers used in BIP Index (see Results for a list of variables and category constituents):

(1) Human indicators

a. Human welfare and social development indicators: DVH

b. Economic development indicators: DVE

c. Information technology indicators: ICT

d. Resource availability and power indicators: PWR

e. Financial power indicators: PWF

(2) Environmental indicators

a. Biological diversity data indicators: BIO

b. Ecological, environmental and human impact indicators: ENV

(3) Intrinsic indicators

a. Physical characteristics of country: GEO

b. Population size and features: POP

3,695 variables were identified as related to the development of countries or societies in category 1, which can be described as 'human indicators', dependent on human development. In addition, 202 variables related specifically to the technical infrastructure needed for informatics development. 1,093 variables were identified in category 2. Some of these may have been influenced by human development, but on themselves may evolve independently. Collectively, they describe the 'environment' that may in turn drive (or compose) biodiversity and therefore be related to the existence of data, irrespective of whether the data have been discovered or not. Category 3 includes variables related to the 'size' or 'weight' (such as area, GDP, or population) of the country that can be used to relativize other variables. 95 variables belonged to category 3.

Some of the variables were in turn composite indexes or ranks calculated from other variables. The main sources for these potential indicator variables were:

• The Food and Agriculture Organization of the United Nations [7]

• The Global Biodiversity Information Facility [8]

• The Global Footprint Network [9]

• The International Telecommunications Union [10]

• The International Union for the Conservation of Nature [11]

• The Legatum Institute [12]

• The New Economics Forum [13]

• The United Nations Development Program [14]

• The United Nations Environment Program [15]

• The World Bank's World Development Index Database [16]

• The World Resources Institute [17]

• The World Values Survey Network [18]

Furthermore, response (biodiversity informatics) data were also collected, including literature, meta-analyses of GBIF data, and results from at least two Task Group provisional reports: the Content Needs Assessment (CNA) Task Group (AHA, VC, and DP Faith, personal communication) and the Global Strategy and Action Plan for Mobilization of Natural History Collections Data (GSAP-NHC) Task Group [5].

### Dataset collection and data organization

Most variables were collected from the sources through organized queries, or in some cases digitized from semi-digital sources. Whenever possible or available, time series were collated as selected annual data. The time span ranged from 1990 to the latest available data, with a majority of series including data from 1990, 1995, 2000, and all the years in the 21st century up to 2008 or even 2009 for a few variables. In all, the collection included some 36,700 annual datasets under scrutiny.

As the different sources provide data in different formats, all data have to be compiled into a manageable data format. A database was constructed with a common field structure to accommodate data from disparate sources in a way amenable to analysis. The table-like sources were converted into a vector file, where each record was an individual datum with attributes relating its source, type, variable name, year, and country. This file, containing over 4 million records for primary (not derived) variables, including missing values, became the base source.

The next step was to reorganize the data into time series and variables. From the base source, tables of country versus latest available variable (or country versus year versus variable) were produced as needed and a working file containing the latest available data from selected variables for each country, as well as the derivative variables, was created. This 800,000-record table was the one effectively subjected to statistical analysis (Figure 3) and is available online as a CSV file [19].

### Variable selection, normalization and substitution

Although the constructed database contained country- and year-specific data that theory suggested could have had some meaning (either known or potential) for the drivers or dimensions of the BIP Index, there was no point in including too many variables in the index. If there were too many missing values, for instance, meaningful inference could be prevented. Besides, the purpose of the BIP Index was not only to predict biodiversity informatics capacity, but also to provide some insight on what factors were important and what were not. Therefore, an initial filtering of variables was made by discarding those not significantly correlated with at least one of the dimensions (Figure 2).

As a majority of variables and all response variables showed non-normal distributions, and many resisted statistical renormalization attempts, Spearman's rank correlation was chosen to discard both variables with non-significant correlations and significant variables with Spearman's rank correlation coefficient < 0.5 ('low-response' variables). Correlations were made pair-wise, using all possible data pairs for each pair predictor-response. About 50% of the variables were thus discarded. The remaining variables were replaced by their ranks and normalized (rescaled) to lie between 0 (lowest rank of the set) and 1 (highest rank); the normalization was of the type:

*x*(*n*) = [*X* - *X*(*min*)]/[*X*(*max*) - *X*(*min*)]. (1)

The surviving variables proceeded further to analysis, each within its own driver. The number of variables that each driver contained varied according to variable availability, but many were also discarded at a later stage. The initial, 'agnostic' composition of the drivers is shown in Table 1.

**Table 1:** Initial number of predictor variables

Driver	Variables
DVH	122
DVE	78
ICT	28
PWR	58
PWF	257
BIO	48
ENV	55
GEO	6
POP	9

Number of predictor variables within each driver that have Spearman rank correlations higher than 0.5 with at least one of the response variables.

A known problem in correlating a set of predictor variables with a set of response variables is the effect of high correlations between predictors that may appear, lending these predictors undue weight. In multiple regression models, this is known as collinearity [20]. To remove this effect, highly correlated predictor variables were substituted by a composite created from a principal components analysis (PCA) [21], which was also tested by regression against the response variables.

The missing values for the variables were also a cause of major concern. The prevalence of missing data forced the index to use available data only, rather than the usual sum of components found in common multiple regression models. As Inboden and Streeter [22] explain, ideally all variables contributing to a composite index should have data, as the index would otherwise lack a component. There are three possible approaches to solve this: data imputation (missing data are substituted by a reasonable imputation), flexible indexing (the contribution of each variable to the index for a country is weighed according to the number of variables for which data are available), or discarding the variable. In the BIP Index, variables with excessive missing data were discarded either totally or from the country's index, and imputations were not made, but the indexes were weighted according to the number of variables available for each country. For the final composite BIP Index, a measure of the degree to which the missing variables may have affected the result is provided, and countries with excess missing variables were not issued a BIP Index ranking.

### Response model and parameterization

Multiple regression analyses (MRA) were used to obtain an approximate idea of the degree to which variations in the rank of the predictor variables, for instance number of endemic species, might correlate with variations in the rank of the response variables such as amount of digitally available data. The MRA coefficients thus became the initial parameters of the model, which could also be further adjusted empirically at a later stage (Figure 2).

36 step-wise MRAs were performed for each driver against dimension. Only significantly correlated variables were retained in the model. For each retained variable, the regression coefficient c*_i_* was saved for use in the model as a weight factor for the *i*th variable in the model, *x_i_*.

The collinearity of the retained variables was examined, and the sets of correlated variables not meeting the relative independence criteria (in general, a variable inflation factor of more than four) were taken out for principal components analyses. The factor scores were retained for the first component, and the variables, *w_j_*, were weighted by this factor score, *z_j_*, in each PCA. The PC of each PCA (now a new variable, composed of the weighted collinear variables that were correlated among themselves) were then returned to the regression model and the MRA was recalculated with the retained variables (minus the collinears) and the principal components (PC) of the collinear variables. The corrected regression coefficient (beta) of the PC was also retained as its own weight factor, *c*. All variables (originally in the regression plus the principal components, but not the collinears that had been substituted by their PCs) were then summed, and standardized by dividing by the number of components. In summary, each of the *k* drivers in the *d* dimension, *D_dk_* was(2)

where *x_i_* is each of *s* variables used directly in the driver, *w_aj_* is each of the *n* correlated variables that are replaced by the *j* PC, *z_aj_* is the weight assigned to *w_aj_* within the *j* PC, and *c_i_*, *c_j_* are the regression coefficients of the variables or PC against response variables.

**Figure 4:**
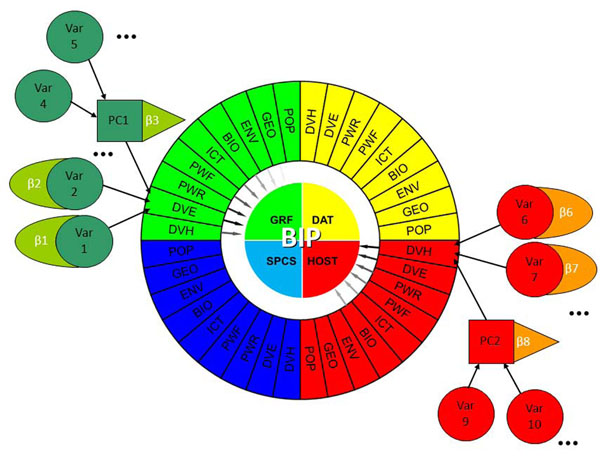
Example and schematics of the driver and dimensions within BIP Index. Colors represent the four dimensions in the BIP index; abbreviations correspond to the drivers behind each dimension (see text). Each driver is a composite of a number of variables, or PCA scores for collinear variables, weighted by their MRA coefficients. Drivers composing each dimension are in turn weighted by a coefficient obtained from linear programming. The BIP Index for a country is the Euclidean distance from the origin along the axes defined by the dimensions (not shown. β, corrected correlation coefficient.

Drivers of predictor variables were statistically matched to proxies or response variables for countries where both sets of data were available. Therefore, each dimension of the BIP Index could be predicted by a set of drivers; each driver, in turn, was composed of a small set of predictor variables retained after MRA and PCA (Figure 4, Table 2; see Results for a list of variables).

**Table 2:** Final number of predictor variables

	Driver								
**Dimension**	**BIO**	**DVH**	**GEO**	**ICT**	**POP**	**PWF**	**PWR**	**ENV**	**DVE**

**DAT**	9	6	4	5	4	7	7	6	3
**GRF**	7	2	5	5	5	6	5	7	2
**HOST**	15	8	3	4	5	6	6	5	1
**SPCS**	6	5	3	3	5	10	5	4	4

Number of variables within each driver retained after MRA and PCA for each dimension (any one predictor may be retained for more than one dimension).

To each driver for each dimension, a coefficient *f_dk_* was given to weight the driver within the final BIP Index: a higher coefficient would mean a higher importance of that driver in that dimension, relative to other drivers in the same dimension. For instance, if the coefficient for driver DVH was low for dimension GRF, that would mean that DVH variables would have little impact on the GRF capacity. Although in theory the selection of this coefficient could be arbitrarily based in judgment, in the BIP Index the drivers' coefficients were found by linear programming (LP) so as to obtain the highest possible correlation between the drivers and the response variables.

The initial, seed values of the coefficients for the LP optimization process were those of the MRA coefficients for each driver. Drivers were combined and the resulting BIP Index dimension was tested against the corresponding response variable: for instance, all nine drivers for DAT were weighted by their coefficients (resulting from the corresponding MRA), and then these coefficients *f_dk_* were made to fluctuate in a Monte Carlo loop by random walk. On each loop, the correlation coefficient was reevaluated and the new values of *f_dk_* were retained if they increased. The loop was repeated until no improvement was observed in the correlation coefficient.

Once the coefficients for drivers were found by LP (each driver, in turn, being a combination of predictor variables or PCA scores of variables), a BIP Index dimension was found as an average of drivers available for such dimension.

The final BIP Index score, used to rank the countries, was a combination of the four predicted dimensions *M*, obtained by weighted Euclidean distance of SPCS, HOST, and the weighted average of GRF and DAT. To attribute relative importance to each dimension, another coefficient *e_a_* was applied to each dimension. This coefficient was entirely arbitrary and based solely on expert judgment, and actually constitutes a tuning factor for BIP Index that allows it to stress any of the concept groups in it: data generation, or data hosting. Although we have judged the four dimensions as shown below (see 'Overall BIP Index'), stressing data publishing and intrinsic biodiversity potential more than raw data generation capacity, other uses of BIP Index may seek to rank countries according to this capacity using appropriate *e_a_* coefficients.

### Final formulation

The final formulation of BIP Index is as follows:(3)

where(4)

and *D_dk_* is as in equation (2).

## Results

### List of variables in the BIP Index model

Additional file 1 shows the set of variables selected by rank correlation, MRA and PCA for each driver in each dimension. Beta is the corrected regression coefficient for the variable, or the PCA score on component 1 of the corresponding PCA. (In the model, PCA scores have been transformed to percentages of PCA scores; they should not be compared directly with regression coefficients for the raw variables.) Coefficients are applicable to the standardized ranks of variables.

### Parameters of the model

**Figure 5:**
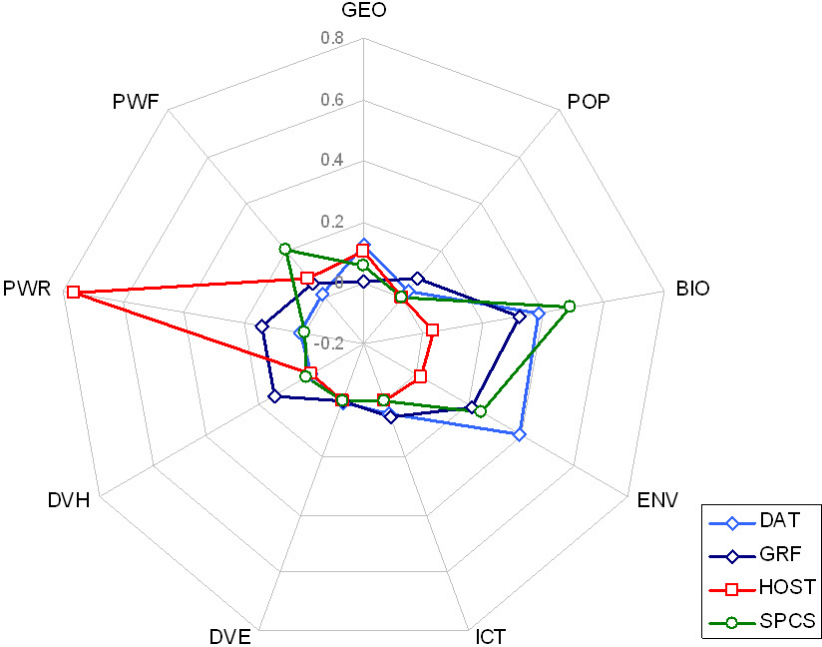
Relative importance of drivers (radii) when predicting BIP dimensions (plot lines). The further from the center of the plot, the more important that driver is for the dimension. For example, the hosting capacity (HOST dimension) seems related mostly to the set of variables depicting the economical and resource power of countries (PWR driver). DAT: raw availability of data; GRF: Availability of quality (geo-referenced) data; HOST: hosted data; SPCS: species richness of generated data. GEO, POP: drivers of general variables; BIO: biological and species data; ENV: environmental indicators; ICT: information technology indicators; DVE, DVH: human and social development indicators; PWR, PWF: economical and financial power.

Table 3 shows the coefficients for the drivers optimized after LP. Their relative importance across the dimensions can be seen in Figure 5, where the coefficients have been normalized for comparison. HOST is highly reliant on PWR (resources and energy available to the country), whereas the data generation dimensions are much more dependent on biological or environmental drivers. It is noteworthy that the biological richness driver (SPCS) is the one most reliant on biological variables, and that some drivers had no significance for certain dimensions in the model once optimized.

**Table 3:** Table of coefficients

	Driver								
**Dimension**	**GEO**	**POP**	**BIO**	**ENV**	**ICT**	**DVE**	**DVH**	**PWR**	**PWF**

DAT	6.37	1.34	19.45	19.7	2.39	0.06	0.02	0.8	0.77
GRF	0.22	3.8	15.97	10.64	2.63	0	6.84	6.79	3.04
HOST	2.14	0	0.72	0.42	0	0	0	15.78	1.66
SPCS	1.65	0	14.81	7.32	0	0	0.57	0	6.04

*f_dk_* coefficients for each driver in each dimension optimized after LP. Zero coefficients lead to the driver concerned being discarded from the dimension. For example, POP driver weights only in the DAT and GRF dimension, and SPCS is dependent only on variables in the GEO, BIO, ENV, DVH and PWF drivers

### Adjustment of the model

**Figure 6:**
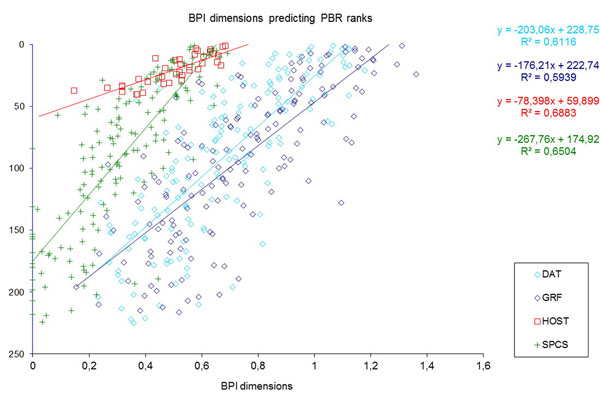
Scatter plots of calculated BIP Index (BPI) dimensions against the corresponding ranked response variables (GBIF statistics), for the cases where these data exist. HOST data exist only for participant countries. Regression coefficients are only indicative.

The model coefficients were obtained from the set of countries for which data existed for all response variables, that is, countries hosting data in GBIF indexes. The predicted BIP Index dimensions are plotted against the actual rank of the countries according to the response variables (DAT, GRF, HOST, SPCS). For HOST, only countries already providing data can be plotted. The adjustment seems good in all cases (Figure 6) but not all countries could be plotted, as some lacked enough data and fell below a quality threshold, arbitrarily defined as the country having data for at least 75% of the variables used in the dimension.

### Overall BIP Index

The overall BIP Index for a country has been defined as the average Euclidean distance to the origin of the dimensions in the BIP Index (DAT, GRF, HOST, SPCS). In the current formulation of the BIP Index, these dimensions have been assigned the following coefficients: DAT: 0.1; GRF: 0.2; HOST: 0.4; SPCS: 0.3.

Therefore, a country is a point in a four-dimensional space, the dimensions being the four BIP Index components multiplied by their importance coefficients.

**Figure 7:**
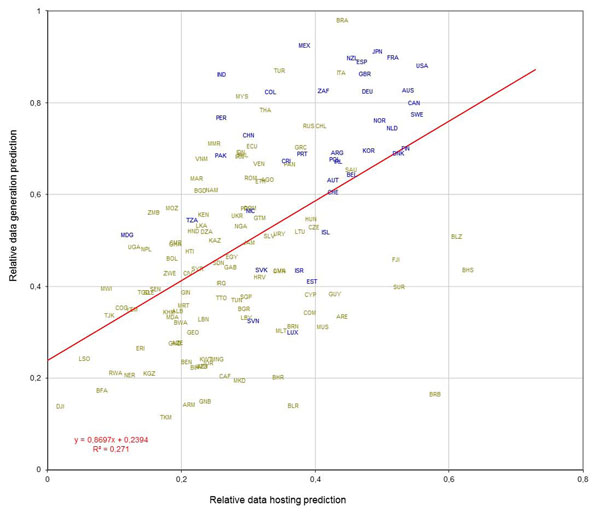
Scatter plot of relative data hosting prediction (HOST dimension versus relative data generation capacity, combining SPCS, DAT and GRF dimensions, for each country. ISO alpha-3 country codes. Blue: Countries participating in GBIF at the time of analysis.

However, for analytical purposes the number of dimensions can be reduced. By averaging DAT and GRF dimensions into one single DAT-GRF dimension, a country can then be represented as a point in common three-dimensional space, whose coordinates are those of the three remaining dimensions (DAT-GRF, HOST, SPCS). Points further from the origin thus have the highest BIP Index. Furthermore, the remaining two dimensions related to biodiversity generation data can be merged into one for examination purposes, which combines 'abundance' of biodiversity data (DAT-GRF) with its 'richness' (SPCS), resulting in a kind of mimic of biological diversity that represents two of the concepts in BIP Index summarized as the data generation capability. This mimic can be plotted in two-dimensional space against the data hosting capability (Figure 7). The regions of interest, naturally, would be the extremes of the plot. The highest extreme represents countries with high data generation capacity and high data hosting capacity, some of which are currently not sharing their data through GBIF but could eventually become highly significant partners if they joined the network.

The plot also shows the potential for data share equalization. Countries in the bottom right region of the plot are not likely to produce many data, but could host data from large potential data-generator countries in the top left part of the plot that may lack this capacity.

### BIP Index ranking

**Figure 8:**
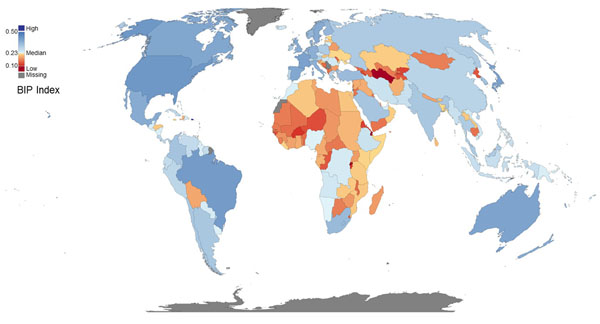
Map representing the BIP Index of all countries for which it could be calculated.

Countries can be ranked according to their BIP Index, calculated according to the methodology explained here (Table 4, Figure 8). Not all countries have data for all required variables in the BIP Index. Therefore, an indicator of reliability has been devised based on the relative number of variables in BIP Index for which data are available for a country (relative reliability score, RRS). A threshold of 75% has been established, and countries for which more than 25% of the variables are missing are not eligible to be included in this version of the BIP Index.

**Table 4:** Rank of selected countries according to their Biodiversity Informatics Potential Index

Rank	Country	ISO	BIP Index	RRS (%)
**1**	**UNITED STATES**	**USA**	**0.4298**	**97**
2	BRAZIL	BRA	0.4211	94
**3**	**FRANCE**	**FRA**	**0.4123**	**98**
**4**	**JAPAN**	**JPN**	**0.4108**	**99**
**5**	**AUSTRALIA**	**AUS**	**0.4069**	**96**
**6**	**CANADA**	**CAN**	**0.3996**	**97**
**7**	**SPAIN**	**ESP**	**0.3981**	**99**
**8**	**SWEDEN**	**SWE**	**0.3930**	**98**
**9**	**NEW ZEALAND**	**NZL**	**0.3928**	**94**
**10**	**MEXICO**	**MEX**	**0.3909**	**100**
**11**	**UNITED KINGDOM**	**GBR**	**0.3886**	**97**
**12**	**GERMANY**	**DEU**	**0.3840**	**98**
13	ITALY	ITA	0.3832	98
**14**	**NETHERLANDS**	**NLD**	**0.3730**	**97**
**15**	**NORWAY**	**NOR**	**0.3692**	**93**
**16**	**FINLAND**	**FIN**	**0.3689**	**97**
**17**	**DENMARK**	**DNK**	**0.3620**	**98**
**18**	**SOUTH AFRICA**	**ZAF**	**0.3610**	**97**
19	TURKEY	TUR	0.3585	98
*20*	*BELIZE*	*BLZ*	*0.3509*	*82*
**21**	**KOREA, REPUBLIC OF**	**KOR**	**0.3443**	**94**
**22**	**COLOMBIA**	**COL**	**0.3439**	**100**
*23*	*BAHAMAS*	*BHS*	*0.3414*	*77*
*24*	*SAUDI ARABIA*	*SAU*	*0.3395*	*87*
**25**	**INDIA**	**IND**	**0.3354**	**94**
**26**	**ARGENTINA**	**ARG**	**0.3353**	**97**
**27**	CHILE*	**CHL**	**0.3349**	**99**
28	RUSSIAN FEDERATION	RUS	0.3301	93
29	MALAYSIA	MYS	0.3284	92
**30**	**BELGIUM**	**BEL**	**0.3278**	**96**
**31**	**POLAND**	**POL**	**0.3264**	**99**
32	THAILAND	THA	0.3258	96
**33**	**IRELAND**	**IRL**	**0.3249**	**96**
**34**	**AUSTRIA**	**AUT**	**0.3174**	**96**
35	GREECE	GRC	0.3148	99
**36**	**SWITZERLAND**	**CHE**	**0.3130**	**93**
**37**	**CHINA**	**CHN**	**0.3101**	**96**
**38**	**PORTUGAL**	**PRT**	**0.3089**	**97**
**39**	**PERU**	**PER**	**0.3057**	**98**
**40**	**COSTA RICA**	**CRI**	**0.3031**	**98**
*41*	*FIJI*	*FJI*	*0.3015*	*78*
42	PANAMA	PAN	0.2996	97
43	VENEZUELA	VEN	0.2963	95
44	ECUADOR	ECU	0.2947	99
*45*	*SURINAME*	*SUR*	*0.2927*	*77*
*46*	*BARBADOS*	*BRB*	*0.2908*	*76*
47	INDONESIA	IDN	0.2898	94
48	PHILIPPINES	PHL	0.2885	97
*49*	*MYANMAR*	*MMR*	*0.2854*	*77*
**50**	**PAKISTAN**	**PAK**	**0.2840**	**98**
51	ETHIOPIA	ETH	0.2831	91
52	IRAN (ISLAMIC REPUBLIC OF)	IRN	0.2829	93
53	CZECH REPUBLIC	CZE	0.2768	92
**54**	**ICELAND**	**ISL**	**0.2759**	**93**
55	HUNGARY	HUN	0.2741	96
*56*	*ANGOLA*	*AGO*	*0.2721*	*83*
57	VIET NAM	VNM	0.2717	94
*58*	*ROMANIA*	*ROM*	*0.2676*	*76*
59	DOMINICAN REPUBLIC	DOM	0.2639	97
60	PARAGUAY	PRY	0.2636	94
61	GUATEMALA	GTM	0.2634	95
*62*	*GUYANA*	*GUY*	*0.2610*	*76*
63	LITHUANIA	LTU	0.2598	91
*64*	*UNITED ARAB EMIRATES*	*ARE*	*0.2594*	*79*
65	NICARAGUA	NIC	0.2574	95
66	MOROCCO	MAR	0.2553	99
67	URUGUAY	URY	0.2539	97
***68***	***ISRAEL***	***ISR***	***0.2538***	***87***
69	EL SALVADOR	SLV	0.2510	91
*70*	*NIGERIA*	*NGA*	*0.2506*	*87*
71	NAMIBIA	NAM	0.2478	91
72	BANGLADESH	BGD	0.2414	92
**73**	**ESTONIA**	**EST**	**0.2409**	**93**
74	UKRAINE	UKR	0.2389	94
75	JAMAICA	JAM	0.2386	92
*76*	*CYPRUS*	*CYP*	*0.2375*	*89*
77	KENYA	KEN	0.2364	96
*78*	*OMAN*	*OMN*	*0.2306*	*90*
*79*	*KAZAKHSTAN*	*KAZ*	*0.2303*	*87*
**80**	**TANZANIA**	**TZA**	**0.2289**	**93**
*81*	*MAURITIUS*	*MUS*	*0.2280*	*85*
*82*	*COMOROS*	*COM*	*0.2276*	*75*
83	HONDURAS	HND	0.2274	97
84	ZAMBIA	ZMB	0.2273	91
*85*	*MOZAMBIQUE*	*MOZ*	*0.2262*	*89*
86	LATVIA	LVA	0.2256	95
87	ALGERIA	DZA	0.2249	95
88	EGYPT	EGY	0.2240	97
**89**	**SLOVAKIA (SLOVAK REPUBLIC)**	**SVK**	**0.2228**	**91**
*90*	*BRUNEI DARUSSALAM*	*BRN*	*0.2220*	*79*
***91***	***LUXEMBOURG***	***LUX***	***0.2191***	***86***
92	SRI LANKA	LKA	0.2187	97
*93*	*CROATIA*	*HRV*	*0.2164*	*90*
94	SUDAN	SDN	0.2123	93
*95*	*GABON*	*GAB*	*0.2098*	*88*
96	SINGAPORE	SGP	0.2064	90
97	GHANA	GHA	0.2045	97
*98*	*MALTA*	*MLT*	*0.2044*	*84*
99	SYRIAN ARAB REPUBLIC	SYR	0.2044	91
*100*	*IRAQ*	*IRQ*	*0.2037*	*82*
101	CAMEROON	CMR	0.2028	96
*102*	*HAITI*	*HTI*	*0.2028*	*82*
103	TUNISIA	TUN	0.2018	93
104	BOLIVIA	BOL	0.1992	96
*105*	*COTE D'IVOIRE*	*CIV*	*0.1961*	*89*
*106*	*NEPAL*	*NPL*	*0.1960*	*88*
**107**	**SLOVENIA**	**SVN**	**0.1956**	**92**
*108*	*BELARUS*	*BLR*	*0.1922*	*87*
*109*	*UGANDA*	*UGA*	*0.1903*	*81*
110	BULGARIA	BGR	0.1900	97
111	TRINIDAD AND TOBAGO	TTO	0.1897	90
***112***	***MADAGASCAR***	***MDG***	***0.1892***	***87***
*113*	*BAHRAIN*	*BHR*	*0.1888*	*78*
*114*	*ZIMBABWE*	*ZWE*	*0.1867*	*88*
*115*	*LIBYAN ARAB JAMAHIRIYA*	*LBY*	*0.1847*	*78*
*116*	*GUINEA*	*GIN*	*0.1831*	*82*
*117*	*MACEDONIA*, *THE FORMER YUGOSLAV REPUBLIC OF*	*MKD*	*0.1672*	*83*
*118*	*SIERRA LEONE*	*SLE*	*0.1667*	*78*
*119*	*MONGOLIA*	*MNG*	*0.1656*	*88*
120	BOTSWANA	BWA	0.1649	91
*121*	*TOGO*	*TGO*	*0.1643*	*87*
122	LEBANON	LBN	0.1641	92
*123*	*MAURITANIA*	*MRT*	*0.1636*	*76*
*124*	*UZBEKISTAN*	*UZB*	*0.1631*	*80*
125	SENEGAL	SEN	0.1625	98
*126*	*CENTRAL AFRICAN REPUBLIC*	*CAF*	*0.1606*	*76*
*127*	*KUWAIT*	*KWT*	*0.1598*	*81*
128	JORDAN	JOR	0.1583	94
129	YEMEN	YEM	0.1580	90
130	CAMBODIA	KHM	0.1562	91
131	ALBANIA	ALB	0.1526	92
*132*	*MALI*	*MLI*	*0.1524*	*80*
*133*	*BENIN*	*BEN*	*0.1521*	*90*
*134*	*GEORGIA*	*GEO*	*0.1508*	*89*
*135*	*BOSNIA AND HERZEGOVINA*	*BIH*	*0.1491*	*78*
*136*	*MALAWI*	*MWI*	*0.1485*	*83*
*137*	*GAMBIA*	*GMB*	*0.1469*	*80*
*138*	*MOLDOVA*, *REPUBLIC OF*	*MDA*	*0.1450*	*87*
*139*	*AZERBAIJAN*	*AZE*	*0.1417*	*89*
140	CONGO	COG	0.1337	91
*141*	*TAJIKISTAN*	*TJK*	*0.1309*	*86*
*142*	*GUINEA-BISSAU*	*GNB*	*0.1293*	*75*
*143*	*NIGER*	*NER*	*0.1246*	*79*
*144*	*ARMENIA*	*ARM*	*0.1215*	*85*
145	KYRGYZSTAN	KGZ	0.1153	90
*146*	*ERITREA*	*ERI*	*0.1134*	*75*
*147*	*BURKINA FASO*	*BFA*	*0.1009*	*79*
*148*	*RWANDA*	*RWA*	*0.0987*	*82*
*149*	*TURKMENISTAN*	*TKM*	*0.0970*	*78*
*150*	*LESOTHO*	*LSO*	*0.0841*	*75*
*151*	*DJIBOUTI*	*DJI*	*0.0575*	*78*

Bold, countries participating in GBIF; italics, countries with a moderate relative reliability score (RRS < 90%), which may affect BIP Index ranking. Countries for which the relative reliability score was lower than 75% are not included. RRS is the percentage of variables required in the BIP Index model that exist for the country (higher is better). ISO: ISO 3166-1-alpha-3 country codes [23]. *Chile became participant country after the BIP Index was calculated, and its dataset was therefore not used in the MRA for the model parameters.

It should be noted once again that the BIP Index is calculated on standardized ranks of the variables. Therefore, relative differences in the BIP Index between countries do not translate into a measure of potential other than for the specific purpose of ranking the countries according to this scale.

## Discussion

To the best of our knowledge, the BIP Index as presented here is the first ever attempt at developing and prototyping a matrix of (a) assessing progress to date, (b) rationalizing future investment and (c) ensuring uniform progress in the field of biodiversity informatics. During the conceptualization and prototyping exercise, we have tried to ensure that all possible parameters and factors that would affect such an index, and for which data could be found, were taken into consideration. Nevertheless, we recognize that arguments can always be put forward in favor of inclusion of some additional factors and omission of some existing ones. Thus, the BIP Index is and will continue to be a complex, evolving exercise. This is mainly because a multitude of factors influence the relevance, robustness and acceptance of such an index. In the future, three key aspects will improve the relevance, robustness and acceptance of BIP Index: (i) validation, (ii) indicator robustness and (iii) increased attention to and investment in biodiversity informatics.

### Validation

This being the first BIP Index, its outcomes and inferences drawn from it need to be tested and verified in biodiversity rich (especially mega-diverse), developing and under-developed regions, as well as data-rich countries. This will help in realizing the relative fitness of the BIP Index, and identifying parameters that will further strengthen the index. It is therefore essential that feedback be received from the stakeholder communities and experts involved in development of similar indices on the significance and usability of such an index, before the next version of the BIP Index. Specific inputs on the methodology adopted, inclusion and/or omission of parameters will be extremely useful in enhancing the robustness and usefulness of the BIP Index.

### Indicator robustness

The present version of the BIP Index has been developed by drawing data from multiple sources. Thus, granularity and temporal scales of these data resources vary from one another. As evident from preceding sections, normalization of such heterogeneous and multi-varied indicators is a daunting task, which makes developing an index of this nature a complex process. During this exercise we felt the need for increased accessibility to key data and parameters that might influence the BIP Index, especially data on the state of the art of biodiversity information and biodiversity informatics in non-GBIF countries, because a mechanism to access such data from these nations is currently lacking. Thus, accessibility to more up-to-date, accurate data on various parameters will help in developing a stable, credible and representative BIP Index.

### Uniform attention and investment in biodiversity informatics

Biodiversity informatics as a scientific discipline is in its relatively early stages, and is not recognized as a mainstream discipline on an equal footing in all regions of the globe. Furthermore, it receives a varied degree of scientific and socio-political attention in different regions. Thus, the global investment in biodiversity informatics is unequal. We believe that outcomes and inferences of the BIP Index will encourage a rationalization and harmonization process of increased yet uniform attention and investment in biodiversity informatics, especially in the regions with high potential to make rapid progress. This will generate more data on parameters that influence BIP Index development and its robustness.

We therefore hypothesize that the relevance, robustness and acceptance of the BIP Index is directly proportional to validation, indicator robustness and attention and investment to biodiversity informatics.

A further issue is our choice of countries as units for developing the BIP Index. Our choice of a 'country-based BIP Index' is intentional because attention and investment in biodiversity informatics is determined and influenced by nations on the basis of several considerations and not by the sub-disciplines, ecosystem focus or priorities.

Finally, there is a need for furthering development and communication of this and subsequent versions of the BIP Index by the GBIF. We believe that GBIF, being the inter-governmental initiative in the area of biodiversity informatics, is the natural venue to support the development of the BIP Index. As GBIF aims to be the foremost global resource for biodiversity information [24], it requires a mechanism and/or instrument to (a) assess the state of the art of biodiversity informatics, (b) suggest the potential of countries to strengthen, advance and benefit from investment in biodiversity informatics, and (c) harmonize global progress in biodiversity informatics. We believe that the BIP Index provides one such comprehensive mechanism that can encourage countries in strengthening, investing and collaborating to ensure that biodiversity information is freely and openly accessible to anyone, anytime and anywhere for the benefit of the science, society and a sustainable future.

## Conclusions

Improved discovery and accessibility of biodiversity data helps to address both scientific and social issues. Furthermore, it is essential for informed decisions for sustainable development of biotic resources and the ecosystems that harbor them. However, this calls for uniform spread and accessibility of such data. Unfortunately, our progress in biodiversity informatics to date is not uniform across the globe. We do not have yet a mechanism to measure our progress in biodiversity informatics that can encourage countries in making demand-driven and deterministic investment in achieving uniform progress in biodiversity informatics. We believe that such uniform progress will help to reduce the existing imbalance of accessibility to biodiversity.

The BIP Index could potentially help in identifying countries most likely to contribute to filling gaps in digitized biodiversity data; assist countries potentially in need (for example mega-diverse countries) to mobilize resources and collect data that could be used in decision-making; and allow identification of which biodiversity-informatics-resourced countries could afford to assist countries lacking in biodiversity informatics capacity.

Further investigations in stabilizing and enriching the BIP Index are essential. Following validation, appropriate parameterization is likely to be essential during the next version of the BIP Index to ascertain or enhance its robustness. This will certainly require a number of iterations of the BIP Index in years to come. Given the political attention and trend of increased investment in biodiversity science, the BIP Index will help in rationalizing such an investment, leading to better understanding of the state and progress in the area of biodiversity informatics. The BIP Index should prove a useful tool for local to global initiatives such as the Intergovernmental Panel on Climate Change (IPCC), the Intergovernmental Science-Policy Platform on Biodiversity and Ecosystem Services (IPBES), the Convention on Biological Diversity (CBD), and Group on Earth Observations Biodiversity Observation Network (GEO-BON). As the BIP Index proves useful in harmonizing the generation, discovery, publishing and accessibility of biodiversity data, it can potentially form an essential mechanism in the science-policy-society interface for biodiversity.

## Competing interests

The authors declare that they have no competing interests.

## Authors' contributions

AHA devised the analytical approach and the BIP model, collated the database, performed the analyses and drafted the manuscript. VC conceived the BIP Index idea and helped to draft the manuscript. NK contributed to crystallizing the concept.

## Supplementary Material

Additional file 1 The following file is available: a list of predictor variables and their coefficients used in each driver retained after MRA and PCA for each dimension (Additional file 1). Click here for file
