# Supplementary material for: The Biodiversity Informatics Potential Index
Source: BMC Bioinformatics. 2011 Dec 15;12(Suppl 15):S4. doi: 10.1186/1471-2105-12-S15-S4 (PMC3287447; doi:10.1186/1471-2105-12-S15-S4)
Supplement: Additional file 1 — The following file is available: a list of predictor variables and their coefficients used in each driver retained after MRA and PCA for each dimension (Additional file 1). [file 1471-2105-12-S15-S4-S1.docx]

**List of predictor variables and their coefficients used in each driver retained after MRA and PCA for each dimension**

*DAT dimension*

| *Driver* | *Variable code* | *Variable name* |  | | *Beta** |
| --- | --- | --- | --- | --- | --- |
| BIO | ANIMS_RED_LIST | Animals, total number in Red List | PC | | 0.9013 |
| BIO | BIOSPHRES_NO.PG | Biosphere Reserves, Number (per US$ GDP) |  | | -0.4202 |
| BIO | CONIF_NO.PG | Conifers and cycads, number of species (per US$ GDP) |  | | 0.16598 |
| BIO | ER.LND.PTLD.TR.NO | Terrestrial protected areas (number) |  | | 0.29831 |
| BIO | MAMM_NO | Mammals, Number of Species | PC | | 0.83958 |
| BIO | PLANTS_RED_LIST | plants, total number in Red List |  | | 0.34293 |
| BIO | RAMSAR_NO | Protected Areas: Wetlands of international importance (Ramsar sites), number | | | 0.28261 |
| BIO | SPECIES_THRTN_IUCN | Total species, threatened | PC | | 0.83426 |
| BIO | VASPL_END | Vascular Plant, Number of Species Endemic | PC | | 0.80389 |
| DVE | ER_MG_CS_PC | Gasoline Consumption (liters per capita) |  | | 0.26876 |
| DVE | IS.ROD.PSGR.K6 | Roads, passengers carried (million passenger-km) |  | | -0.3629 |
| DVE | NE.CON.GOVT.KD | General government final consumption expenditure (constant 2000 US$) | | | 1.04193 |
| DVH | ISO14001 | Certified ISO-14001 companies (number) |  | | 0.31723 |
| DVH | LIFE_SATISF | Life satisfaction (0=low, 10=high) |  | | 0.57175 |
| DVH | SE.PRM.TCHR | Primary education, teachers | PC | | 0.93611 |
| DVH | SE.SEC.TCHR | Secondary education, teachers | PC | | 0.98133 |
| DVH | SH.TBS.INCD | Incidence of tuberculosis (per 100,000 people) |  | | 0.42308 |
| DVH | SH.XPD.EXTR.ZS | External resources for health (% of total expenditure on health) | PC | | -0.4047 |
| ENV | EN.ATM.CO2E.KT | CO_2_ emissions (kt) |  | | -0.464 |
| ENV | ER.H2O.FWIN.ZS | Annual freshwater withdrawals, industry (% of total freshwater withdrawal) | | | 0.17436 |
| ENV | FISH_PROD_MAR | Fishery Production Totals (aquaculture and capture): Marine production, Tm | | | 0.30265 |
| ENV | H2O_POVNDX | Water Poverty Index (Index Number 0-100; lower scores indicate water scarcity and poor water provision) | | | 0.19414 |
| ENV | URBAN_AREA.PC | Urban and built-up areas, kHa (per capita) |  | | 0.2808 |
| ENV | USE_CHEM_WEED | Consumption of weed control chemicals (tonnes) |  | | 0.76579 |
| GEO | AG.SRF.TOTL.K2 | Surface area (km^2^) | PC | | 0.86721 |
| GEO | COASTLINE | Length of coastline (km) | PC | | 0.74688 |
| GEO | H2O_BODIES | Water bodies, km^2^ | PC | | 0.95106 |
| ICT | BX.GSR.CCIS.CD | ICT service exports (BoP, current US$) |  | | 0.49636 |
| ICT | IT.CEL.SETS.PG | Mobile cellular subscriptions (per US$ GDP) | PC | | -0.6983 |
| ICT | IT.CMP.PCMP.PC | Personal computers (per capita) | PC | | 0.87483 |
| ICT | IT.NET.BBND | Fixed broadband Internet subscribers | PC | | 0.88079 |
| ICT | IT.NET.SECR | Secure Internet servers | PC | | 0.91816 |
| POP | SP.POP.0014.TO.ZS | Population ages 0-14 (% of total) | PC | | 0.93518 |
| POP | SP.POP.65UP.TO.ZS | Population ages 65 and above (% of total) | PC | | -0.929 |
| POP | SP.URB.GROW | Urban population growth (annual %) | PC | | 0.91053 |
| POP | SP.URB.TOTL | Urban population |  | | 0.52049 |
| PWF | BM.GSR.NFSV.CD | Service imports (BoP, current US$) | PC | | 0.9834 |
| PWF | DT.DOD.MLTC.CD.PC | PPG, multilateral concessional (DOD, current US$) (per capita) | | | -0.5298 |
| PWF | IC.BUS.NREG | New businesses registered (number) |  | | 0.35818 |
| PWF | IC.IMP.COST.CD.PC | Cost to import (US$ per container) (per capita) |  | | -0.4267 |
| PWF | IS.SHP.GCNW.XQ | Liner shipping connectivity index (maximum value in 2004 = 100) | | | 0.28005 |
| PWF | NV.IND.TOTL.KD | Industry, value added (constant 2000 US$) | PC | | 0.9676 |
| PWF | ST.INT.TVLX.CD | International tourism, expenditures for travel items (current US$) | PC | | 0.97799 |
| PWR | EG.EGY.PROD.KT.OE.PC | Energy production (kt of oil equivalent) (per capita) |  | | 0.16427 |
| PWR | EG.ELC.PROD.KH.PC | Electricity production (kWh) (per capita) | PC | | 0.99339 |
| PWR | EG.USE.CRNW.KT.OE | Combustible renewables and waste (metric tons of oil equivalent) | | | 0.50938 |
| PWR | EG.USE.ELEC.KH.PC | Electric power consumption (kWh per capita) | | PC | 0.99339 |
| PWR | NV.AGR.TOTL.KD.PC | Agriculture, value added (constant 2000 US$) (per capita) | |  | 0.30866 |
| PWR | SL.SRV.EMPL.ZS | Employment in services (% of total employment) | |  | 0.19385 |
| PWR | SL.TLF.TOTL.IN | Labor force, total | |  | 0.16908 |

*PC*: The variable has been taken as a principal component in the formulation. *Beta, corrected regression coefficient

*GRF Dimension*

| *Driver* | *Variable code* | *Variable name* |  | *Beta** | |  |
| --- | --- | --- | --- | --- | --- | --- |
| BIO | BIOSPHRES_NO.PG | Biosphere Reserves, Number (per US$ GDP) |  | | -0.3233 | |
| BIO | ER.BDV.TOTL.XQ | GEF benefits index for biodiversity (0 = no biodiversity potential to 100 = maximum) | PC | | 0.93353 | |
| BIO | ER.LND.PTLD.TR.NO | Terrestrial protected areas (number) |  | | 0.27304 | |
| BIO | MAMM_NO | Mammals, Number of Species | PC | | 0.76584 | |
| BIO | PLANTS_RED_LIST | plants, total number in Red List |  | | 0.39157 | |
| BIO | RAMSAR_NO | Protected Areas: Wetlands of international importance (Ramsar sites), number | | | 0.26449 | |
| BIO | VASPL_END | Vascular Plant, Number of Species Endemic | PC | | 0.91194 | |
| DVE | ER_MG_CS_PC | Gasoline Consumption (liters per capita) |  | | 0.39211 | |
| DVE | NE.CON.GOVT.KD | General government final consumption expenditure (constant 2000 US$) | | | 0.66247 | |
| DVH | LIFE_SATISF | Life satisfaction (0=low, 10=high) |  | | 0.56565 | |
| DVH | SE.PRM.TCHR | Primary education, teachers |  | | 0.56223 | |
| ENV | EE.BOD.TXTL.ZS | Water pollution, textile industry (% of total BOD emissions) |  | | -0.1797 | |
| ENV | ER.H2O.FWTL.K3.PG | Annual freshwater withdrawals, total (billion cubic meters) (per US$ GDP) | | | -0.2925 | |
| ENV | FISH_PROD_TOT | Fishery Production Totals (aquaculture and capture): Total for all species, Tm | | | 0.28122 | |
| ENV | NY.ADJ.DCO2.CD | Adjusted savings: carbon dioxide damage (current US$) |  | | -0.5455 | |
| ENV | NY.ADJ.DMIN.CD.PC | Adjusted savings: mineral depletion (current US$) (per capita) | | | 0.19053 | |
| ENV | URBAN_AREA.PC | Urban and built-up areas, kHa (per capita) |  | | 0.2702 | |
| ENV | USE_CHEM_WEED | Consumption of weed control chemicals (tonnes) |  | | 0.89725 | |
| GEO | AG.SRF.TOTL.K2 | Surface area (km^2^) | PC | | 0.86721 | |
| GEO | COASTLINE | Length of coastline (kilometers) | PC | | 0.74688 | |
| GEO | EEZ | Area of continental shelf up to 200 miles (square kilometers) | | | 0.28175 | |
| GEO | H2O_BODIES | Water bodies, km^2^ | PC | | 0.95106 | |
| GEO | H2O_BODIES | Water bodies, km^2^ |  | | 0.38726 | |
| ICT | DAI | Digital Access Index (0- no access, 100-highest access) | PC | | 0.92544 | |
| ICT | IT.CEL.SETS.PG | Mobile cellular subscriptions (per US$ GDP) | PC | | -0.7681 | |
| ICT | IT.NET.BNDW | International Internet bandwidth (Mbps) | PC | | 0.86012 | |
| ICT | IT.NET.SECR | Secure Internet servers | PC | | 0.9227 | |
| ICT | TX.VAL.TECH.CD.PC | High-technology exports (current US$) (per capita) | PC | | 0.89054 | |
| POP | SP.POP.0014.TO.ZS | Population ages 0-14 (% of total) | PC | | 0.93518 | |
| POP | SP.POP.1564.TO.ZS | Population ages 15-64 (% of total) |  | | -0.458 | |
| POP | SP.POP.65UP.TO.ZS | Population ages 65 and above (% of total) | PC | | -0.929 | |
| POP | SP.URB.GROW | Urban population growth (annual %) | PC | | 0.91053 | |
| POP | SP.URB.TOTL | Urban population |  | | 0.50236 | |
| PWF | BM.GSR.NFSV.CD | Service imports (BoP, current US$) | PC | | 0.97351 | |
| PWF | FI.RES.TOTL.MO.PS | Total reserves in months of imports (per km2) |  | | -0.3339 | |
| PWF | IC.CRD.PRVT.ZS | Private credit bureau coverage (% of adults) |  | | 0.34286 | |
| PWF | NV.IND.TOTL.KD | Industry, value added (constant 2000 US$) | PC | | 0.95905 | |
| PWF | NY.GNS.ICTR.CD | Gross savings (current US$) | PC | | 0.95766 | |
| PWF | ST.INT.RCPT.CD | International tourism, receipts (current US$) | PC | | 0.92129 | |
| PWR | EG.ELC.COAL.KH | Electricity production from coal sources (kWh) |  | | 0.21165 | |
| PWR | EG.ELC.PROD.KH.PC | Electricity production (kWh) (per capita) | PC | | 0.99339 | |
| PWR | EG.USE.CRNW.KT.OE | Combustible renewables and waste (metric tons of oil equivalent) | | | 0.47466 | |
| PWR | EG.USE.ELEC.KH.PC | Electric power consumption (kWh per capita) | PC | | 0.99339 | |
| PWR | NV.AGR.TOTL.KD.PC | Agriculture, value added (constant 2000 US$) (per capita) |  | | 0.32816 | |

*HOST Dimension*

| *Driver* | *Variable code* | | *Variable name* |  | *Beta** | | |
| --- | --- | --- | --- | --- | --- | --- | --- |
| BIO | ANFIB_NO | Anfibians, Number of Species | | PC | | 0.88797 |  |
| BIO | ANIMS_EN | Animals, endangered | | PC | | 0.88688 |  |
| BIO | ANIMS_RED_LIST | Animals, total number in Red List | | PC | | 0.86676 |  |
| BIO | BIOSPHRES_NO.PG | Biosphere Reserves, Number (per US$ GDP) | | PC | | 0.22541 |  |
| BIO | CONIF_NO | Conifers and cycads, number of species | | PC | | 0.38457 |  |
| BIO | CONIF_NO.PG | Conifers and cycads, number of species (per US$ GDP) | |  | | -0.6756 |  |
| BIO | EN.BIR.THRD.NO.PG | Bird species, threatened (per US$ GDP) | | PC | | 0.48416 |  |
| BIO | EN.MAM.THRD.NO.PG | Mammal species, threatened (per US$ GDP) | | PC | | 0.48044 |  |
| BIO | ER.BDV.TOTL.XQ | GEF benefits index for biodiversity (0 = no biodiversity potential to 100 = maxi) | | PC | | 0.90747 |  |
| BIO | ER.LND.PTLD.TR.NO | Terrestrial protected areas (number) | | PC | | -0.1192 |  |
| BIO | FERNS_NO.PG | Ferns, Number of Species (per US$ GDP) | | PC | | 0.51951 |  |
| BIO | MAMM_NO | Mammals, Number of Species | | PC | | 0.88072 |  |
| BIO | PLANTS_RED_LIST | plants, total number in Red List | | PC | | 0.93048 |  |
| BIO | SPECIES_THRTN_IUCN | Total species, threatened | | PC | | 0.92214 |  |
| BIO | WPA_HA | Protected Areas Existing or Projected (surface, Ha) | | PC | | 0.37954 |  |
| DVE | NE.CON.TOTL.KD.PC | Final consumption expenditure (constant 2000 US$) (per capita) | | | | 0.86376 |  |
| DVH | EN.AGR.EMPL.PG | Economically active population in agriculture (number) (per US$ GDP) | | PC | | 0.92809 |  |
| DVH | HDI | Human Development Index | |  | | -0.4867 |  |
| DVH | LIFE_SATISF | Life satisfaction (0=low, 10=high) | | PC | | -0.883 |  |
| DVH | NY.ADJ.AEDU.CD | Adjusted savings: education expenditure (current US$) | |  | | 0.23204 |  |
| DVH | SE.PRM.PRS5.ZS | Persistence to grade 5, total (% of cohort) | |  | | 0.50241 |  |
| DVH | SH.TBS.INCD | Incidence of tuberculosis (per 100,000 people) | | PC | | 0.90846 |  |
| DVH | SH.XPD.EXTR.ZS | External resources for health (% of total expenditure on health) | | PC | | 0.90511 |  |
| DVH | SM.POP.NETM | Net migration | | PC | | -0.5551 |  |
| ENV | CARBON_FP | Carbon footprint (global hectares per capita) | |  | | -0.3834 |  |
| ENV | EN.ATM.NOXE.KT.CE.PC | Nitrous oxide emissions (thousand metric tons of CO2 equivalent) (per capita) | | | | 0.18726 |  |
| ENV | ER.H2O.FWTL.K3.PG | Annual freshwater withdrawals, total (billion cubic meters) (per US$ GDP) | | | | -0.5665 |  |
| ENV | FISH_PROD_TOT | Fishery Production Totals (aquaculture and capture): Total for all species, Tm | | | | 0.41226 |  |
| ENV | URBAN_AREA | Urban and built-up areas, kHa | |  | | 0.76474 |  |
| GEO | AG.SRF.TOTL.K2 | Surface area (km^2^) | | PC | | 0.86721 |  |
| GEO | COASTLINE | Length of coastline (kilometers) | | PC | | 0.74688 |  |
| GEO | H2O_BODIES | Water bodies, km^2^ | | PC | | 0.95106 |  |
| ICT | IT.CEL.SETS.PG | Mobile cellular subscriptions (per US$ GDP) | | PC | | -0.4069 |  |
| ICT | IT.NET.BBND | Fixed broadband Internet subscribers | | PC | | 0.96542 |  |
| ICT | IT.NET.SECR | Secure Internet servers | | PC | | 0.94192 |  |
| ICT | IT.NET.USER | Internet users | | PC | | 0.87517 |  |
| POP | EN.URB.LCTY.UR.ZS | Population in the largest city (% of urban population) | |  | | -0.3333 |  |
| POP | SP.POP.0014.TO.ZS | Population ages 0-14 (% of total) | | PC | | 0.93518 |  |
| POP | SP.POP.65UP.TO.ZS | Population ages 65 and above (% of total) | | PC | | -0.929 |  |
| POP | SP.URB.GROW | Urban population growth (annual %) | | PC | | 0.91053 |  |
| POP | SP.URB.TOTL.IN.ZS | Urban population (% of total) | |  | | 0.30181 |  |
| PWF | BM.GSR.FCTY.CD | Income payments (BoP, current US$) | | PC | | 0.94579 |  |
| PWF | BX.GSR.FCTY.CD | Income receipts (BoP, current US$) | | PC | | 0.95143 |  |
| PWF | IC.IMP.DURS | Lead time to import (days) | | PC | | -0.7022 |  |
| PWF | LP.LPI.TRAC.XQ | Logistics performance index: Ability to track and trace consignments (1=low to 5=high) | | PC | | 0.84303 |  |
| PWF | NY.GNS.ICTR.CD | Gross savings (current US$) | | PC | | 0.89646 |  |
| PWF | NY.TAX.NIND.CD | Net taxes on products (current US$) | | PC | | 0.89992 |  |
| PWR | EG.ELC.HYRO.KH | Electricity production from hydroelectric sources (kWh) | |  | | -0.3114 |  |
| PWR | EG.ELC.PROD.KH.PC | Electricity production (kWh) (per capita) | | PC | | 0.99339 |  |
| PWR | EG.USE.CRNW.KT.OE | Combustible renewables and waste (metric tons of oil equivalent) | | | | 0.53379 |  |
| PWR | EG.USE.ELEC.KH.PC | Electric power consumption (kWh per capita) | | PC | | 0.99339 |  |
| PWR | NV.AGR.TOTL.KD.PC | Agriculture, value added (constant 2000 US$) (per capita) | |  | | 0.46706 |  |
| PWR | SL.SRV.EMPL.ZS | Employment in services (% of total employment) | |  | | 0.54655 |  |

*PC*: The variable has been taken as a principal component in the formulation. *Beta, corrected regression coefficient

*SPCS Dimension*

| *Driver* | *Variable code* | | *Variable name* | | |  | *Beta** | |
| --- | --- | --- | --- | --- | --- | --- | --- | --- |
| BIO | ANIMS_EN | Animals, endangered | |  | 0.18842 | | |  |
| BIO | BIOSPHRES_NO.PG | Biosphere Reserves, Number (per US$ GDP) | |  | -0.3803 | | |  |
| BIO | CONIF_NO | Conifers and cycads, number of species | |  | 0.2412 | | |  |
| BIO | ER.LND.PTLD.TR.NO | Terrestrial protected areas (number) | |  | 0.2626 | | |  |
| BIO | RAMSAR_NO | Protected Areas: Wetlands of international importance (Ramsar sites), number | | | 0.24203 | | |  |
| BIO | VASPL_END.PG | Vascular Plant, Number of Species Endemic (per US$ GDP) | | | 0.30067 | | |  |
| DVE | EA.PRD.AGRI.KD | Agriculture value added per worker (constant 2000 US$) | |  | -0.3072 | | |  |
| DVE | IS.ROD.PSGR.K6 | Roads, passengers carried (million passenger-km) | |  | -0.4877 | | |  |
| DVE | IS.SHP.GOOD.TU.PG | Container port traffic (TEU: 20 foot equivalent units) (per US$ GDP) | | | -0.4694 | | |  |
| DVE | NE.CON.GOVT.KD | General government final consumption expenditure (constant 2000 US$) | | | 1.29746 | | |  |
| DVH | ISO14001 | Certified ISO-14001 companies (number) | |  | 0.55879 | | |  |
| DVH | LIFE_SATISF | Life satisfaction (0=low, 10=high) | |  | 0.58469 | | |  |
| DVH | SH.STA.ACSN | Improved sanitation facilities (% of population with access) | |  | -0.3966 | | |  |
| DVH | SH.TBS.INCD | Incidence of tuberculosis (per 100,000 people) | |  | 0.35261 | | |  |
| DVH | SH.XPD.EXTR.ZS | External resources for health (% of total expenditure on health) | | | -0.234 | | |  |
| ENV | EE.BOD.TOTL.KG.PG | Organic water pollutant (BOD) emissions (kg per day) (per US$ GDP) | | | 0.24224 | | |  |
| ENV | FISH_PROD_MAR | Fishery Production Totals (aquaculture and capture): Marine production, Tm | | | 0.33837 | | |  |
| ENV | NY.ADJ.DCO2.GN.ZS | Adjusted savings: carbon dioxide damage (% of GNI) | |  | -0.4879 | | |  |
| ENV | USE_CHEM_WEED | Consumption of weed control chemicals (tonnes) | |  | 0.48967 | | |  |
| GEO | AG.SRF.TOTL.K2 | Surface area (km^2^) | | PC | 0.86721 | | |  |
| GEO | COASTLINE | Length of coastline (kilometers) | | PC | 0.74688 | | |  |
| GEO | H2O_BODIES | Water bodies, km^2^ | |  | 0.36068 | | |  |
| ICT | IE.ICT.TOTL.CD | Information and communication technology expenditure (current US$) | | PC | 0.9076 | | |  |
| ICT | IT.NET.BNDW | International Internet bandwidth (Mbps) | | PC | 0.93817 | | |  |
| ICT | IT.NET.SECR | Secure Internet servers | | PC | 0.95058 | | |  |
| POP | EN.POP.DNST | Population density (people per km^2^) | |  | -0.1544 | | |  |
| POP | SP.POP.0014.TO.ZS | Population ages 0-14 (% of total) | | PC | 0.93518 | | |  |
| POP | SP.POP.65UP.TO.ZS | Population ages 65 and above (% of total) | | PC | -0.929 | | |  |
| POP | SP.URB.GROW | Urban population growth (annual %) | | PC | 0.91053 | | |  |
| POP | SP.URB.TOTL | Urban population | |  | 0.5189 | | |  |
| PWF | BX.TRF.CURR.CD | Current transfers, receipts (BoP, current US$) | |  | 0.2847 | | |  |
| PWF | DT.DOD.MLTC.CD.PC | PPG, multilateral concessional (DOD, current US$) (per capita) | | PC | -0.1342 | | |  |
| PWF | FI.RES.TOTL.MO.PG | Total reserves in months of imports (per US$ GDP) | |  | -0.37 | | |  |
| PWF | IC.BUS.NREG | New businesses registered (number) | | PC | 0.14891 | | |  |
| PWF | IS.SHP.GCNW.XQ | Liner shipping connectivity index (maximum value in 2004 = 100) | | | 0.15203 | | |  |
| PWF | NE.IMP.GNFS.KD | Imports of goods and services (constant 2000 US$) | | PC | 0.16657 | | |  |
| PWF | NV.IND.TOTL.KD | Industry, value added (constant 2000 US$) | | PC | 0.16203 | | |  |
| PWF | NY.GNS.ICTR.CD | Gross savings (current US$) | | PC | 0.16387 | | |  |
| PWF | ST.INT.TVLX.CD | International tourism, expenditures for travel items (current US$) | | PC | 0.15707 | | |  |
| PWF | TM.VAL.SERV.CD.WT | Commercial service imports (current US$) | | PC | 0.16215 | | |  |
| PWR | EG.ELC.PROD.KH.PC | Electricity production (kWh) (per capita) | | PC | 0.99339 | | |  |
| PWR | EG.USE.CRNW.KT.OE | Combustible renewables and waste (metric tons of oil equivalent) | | | 0.64812 | | |  |
| PWR | EG.USE.ELEC.KH.PC | Electric power consumption (kWh per capita) | | PC | 0.99339 | | |  |
| PWR | NV.AGR.TOTL.KD.PC | Agriculture, value added (constant 2000 US$) (per capita) | |  | 0.20616 | | |  |
| PWR | NV.AGR.TOTL.ZS | Agriculture, value added (% of GDP) | |  | -0.3053 | | |  |

*PC*: The variable has been taken as a principal component in the formulation. *Beta, corrected regression coefficient
